# Internet of Things with Lightweight Identities Implemented Using DNS DANE—Architecture Proposal

**DOI:** 10.3390/s18082517

**Published:** 2018-08-01

**Authors:** Mariusz Kamola

**Affiliations:** NASK Institute, Kolska 12, 01045 Warsaw, Poland; Mariusz.Kamola@nask.pl; Tel.: +48-603-180-166

**Keywords:** TLSA, IoT, identity provider

## Abstract

Domain Name Service (DNS) and its certification related resource records are appealing alternative to the standard X.509 certification framework, in provision of identities for Internet of Things (IoT) smart devices. We propose to also use DNS to store device owner identification data in device certificates. A working demonstration software has been developed as proof of this concept, which uses an external identity provider run by national authorities. As a result, smart devices are equipped with certificates that safely identify both the device and its owner. Hardware requirements make such a framework applicable to constrained devices. It stimulates mutual trust in machine-to-machine and man-to-machine communication, and creation of a friendlier environment for sale, lease, and data exchange. Further extensions of the proposed architecture are also discussed.

## 1. Introduction

While the rollout of the Internet of Things (IoT) is gaining its momentum, with so many new kinds of devices being equipped with data processing capabilities, one may find strange the fact that in practice, most of those things need some centralized service to interact properly, which is contrary to the original IoT vision. Thermostats and light bulbs, coffee makers, smart buttons, and even smart mains sockets—most of them require their avatar representation in the manufacturer’s cloud, or at least use a proprietary communication protocol. This effectively prevents direct machine-to-machine interaction, also referred to as edge or fog computing [1]. One of the reasons for such a situation is that those small, but intelligent devices are connected with the Internet using a multitude of protocols and communication paths, which are often subject to additional address translation. Application of IPv6 as the common addressing scheme is therefore impossible.

Although, authors believe there is more to this. By keeping their devices in proprietary information silos, providers of IoT management platforms evade the issue of worldwide identity and authenticity management of “things”, and this hinders true fog computing. To make fog computing possible, all devices would have to use Public Key Infrastructure (PKI), which is now the only global authentication technology for Internet communications. Public key infrastructure relies on a proper certification chain all the way down to each single service or device, and is implemented with the use of X.509 certificates [2]. An essential role of an X.509 certificate is to bind human-readable identification data of the certificate owner, that is, user name, with the computer-readable public key the owner utilizes to sign his artifacts (services, documents). The certificate contains many additional fields of technical significance, like Common Name (CN) and validity period. An important addition made in version 3 are optional extension fields, where more descriptive information about the owner can be stored.

Certificates are issued and signed by Certification Authorities (CAs), which follow their identity recognition procedure for certificate owners. Such an approach is hard to imagine for IoT because of the following: (a) certificate price and reliability, (b) management inconvenience for individual owners of smart devices, and (c) requirement that a public IP address be assigned for each device. In particular, there are over 1000 CAs trusted by default by most Internet browsers; if only one of them goes corrupt, it is capable of issuing any number of fake certificates. Getting such a certificate is the first step in man-in-the-middle attack scenarios, resulting in browser redirection to malicious websites.

**Figure 1:**
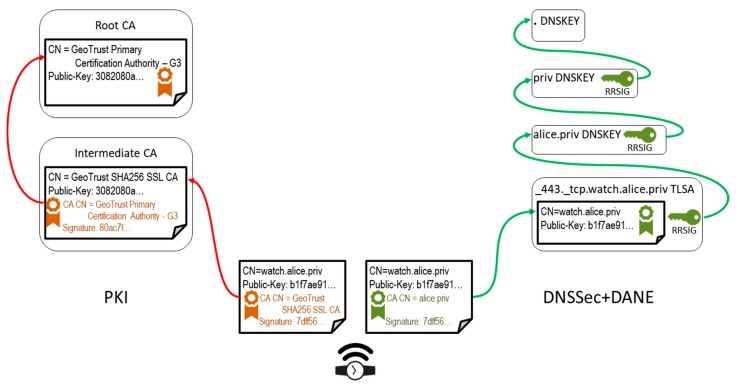
Chains of trust for X.509 certificate presented by a smart device, e.g., a wristwatch. (**Left**): conventional Public Key Infrastructure (PKI) approach; certificate signatures get verified recursively until some root Certification Authorities (CA) is reached, whom a client trusts. (**Right**): secure DNS (DNSSec) + Domain Authenticated Naming services Extension (DANE) approach; device certificate is signed by a self-signed domain owner certificate—but all domain records can be trusted because they are signed with zone signing keys and chained up the hierarchy by key signing keys, up to the root zone (trusted by default). TLSA—Transport Layer Security Authentication.

This PKI vulnerability has been confirmed by real incidents [3] and has become one of the reasons for the introduction of secure DNS (DNSSec) with Domain Authenticated Naming services Extension (DANE) [4,5]. DNSSec + DANE reinforces the traditional PKI, but it also may be used as the replacement for PKI CAs, see Figure 1. In the latter case, domain owners issue certificates for all entities in their domains. In the example in Figure 1, Alice would first create a self-signed certificate for her domain, alice.priv, and use it to make and sign a certificate for her wristwatch. That certificate gets installed in her IoT device, and is also placed in a Transport Layer Security Authentication (TLSA) resource record associated with a unique domain name assigned to that device. All resource records are signed by a domain owner; this, combined with the mechanism of assigning key pairs to domain owners down the DNS hierarchy, creates an unbroken trust chain up to the root domain, whose certificate is trusted globally. Such an alternative architecture for Transport Layer Security (TLS) communication protocols has been demonstrated to work in practice [3]: a web browser plugin has been implemented that performs web page X.509 certificate checks against DANE records, which is all it takes to verify website authenticity.

The appropriateness of DNSSec + DANE application for IoT authentication and relationship management has already been recognized and emphasized by DNS experts and officials [6]. Use of the framework boils down to comparing X.509 certificate emitted by the IoT device with TLSA record for that device. Such a simple scheme, when combined with other DNS capabilities, enables many other communication scenarios. A set of patents by VeriSign Inc., Reston, VA, is based on the common idea of utilizing DNS as a directory for authentication, relationship management, and ontological description of IoT devices.

In particular, patents [7,8] introduce a concept of IoT identity and relationship service that stores the IoT device public key in TLSA record or its equivalent meant to use for e-mail messages authentication. This allows device-to-device secure communication worldwide over untrusted channels, for example, message queues. Additionally, the IoT service manages authorization of the device and of attribute files that contain descriptions of ownership(s) and the device parameters. Such a solution, although incompatible with the work of [5], effectively addresses hardships of massive certification and relationship management of interacting smart devices.

This vision gets developed further in patent [9] where IoT devices expose their communication endpoints via a software component, termed a container. The patent introduces communication and service discovery endpoints for containers. Additionally, establishing data access policies in containers is envisioned, which is possible because communicating parties’ identities are publicly stored in DANE. Authentication, communication, and search capabilities mentioned above are complemented with ontological device annotation and search capabilities, proposed in patent [10]. However, the proposed semantic search process does not explicitly involve DNS.

An alternative to the patented proposals [11] presents a vision of a global IoT device directory accomplished by a dedicated domain name, or several names. This vision is a good example of many other similar research ideas aimed to employ DNSSec and DANE to manage IoT infrastructure. Specifically, the paper suggests that device ontological description be provided autonomously by the device itself, and not stored in DNS. The authors propose to use Extended Environments Markup Language (EEML) and Web Application Description Language (WADL) for this purpose. Device lookup in DNS database would be provided by DNS-based Service Discovery (DNS-SD).

The above initiatives delegate the tasks of device certification and relationship management to DNS instead of IoT management platforms. Yet they skip over the question of how the identity of the IoT device owner be verified by the organization responsible for management of the domain that is about to host that device. The solution is trivial only if the organization and the device owner are the same entity; in such a case, one believes that organization identity has been verified during the process of generation DNSSec key signing keys. Such an approach is still unreliable and impractical. First, credibility of the domain owner identity depends on means used by the registrar in the domain registration process, which are impossible to control worldwide, thus they are not uniform and are sometimes even unknown. Second, to comply with the rules of communication defined in the literature [5], each domain owner must be capable of generating a self-signed certificate, as well as certificates for his/her things.

**Figure 2:**
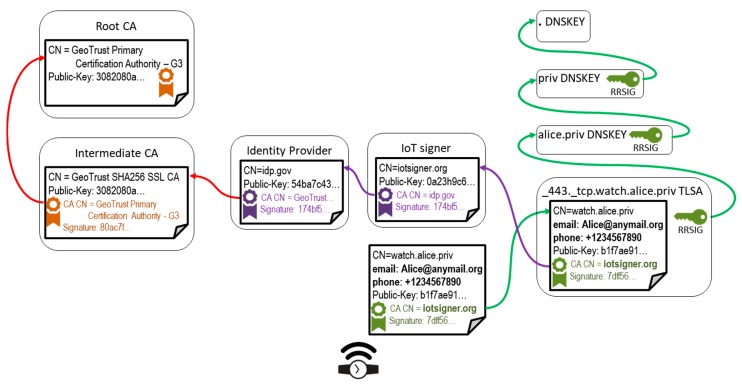
Introduction of identity provider and Internet of Things (IoT) signer services leads to reinforcement of device owner identification and to device certificate generation outsourcing, respectively. Unlike in Figure 1, the device certificate gets signed by the IoT signer, which in turn is authorized to use identity provider services to identify the device owner.

Our contribution addresses both drawbacks. We propose to involve two other entities in the process of certificate generation for an IoT device, as shown in Figure 2. The architecture preserves the domain maintenance role for the device owner, and outsources certificate generation to an external service, the IoT signer, operated by some entity authorized to use Identity Provider (IdP) services in order to authenticate the device owner. The IoT signer service is authorized to retrieve extra data about the device owner, like phone number, e-mail address, or IdP login name; selected data get embedded as X.509v3 extensions into the device certificate being released. Notably, other existing trust chains are essentially unaffected (green and red arrows in Figure 1 and Figure 2). Such an architecture addresses the existing inconveniences of DNSSec + DANE: the domain registrar and owner are relieved from device owner identity check (which is delegated to IdP); also, the device owner is relieved from the certificate generation task (delegated to IoT signer).

Advantages of such an architecture over previous approaches are manifold: (a) a device can be registered in any domain, as well as simultaneously; (b) device certificate can be issued by any organization allowed to use IdP services; and (c) many alternative (or supplementary) IdP services can be used.

Our another contribution is to implement the IoT signer service, and to make an adequate configuration of the rest of the elements of such an architecture to assemble a working demonstrator. Technical implementation details and applicability of the architecture to IoT devices are provided in Section 2. Performance evaluation and possible application scenarios are presented in Section 3. In Section 4, we discuss extensions of the architecture.

## 2. Materials and Methods

**Figure 3:**
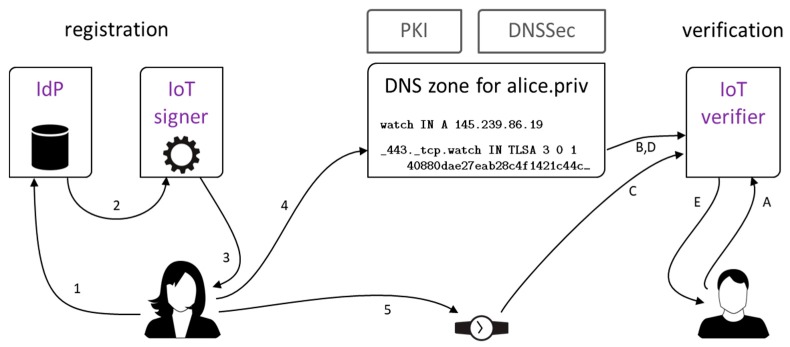
Identity registration scenario (**left**) and identity verification scenario (**right**) for an IoT device. IdP—identity provider.

Roles of the IdP and IoT signer entities are fulfilled by web services available for unrestricted public use, as shown in Figure 3. Together with standard PKI and DNSSec + DANE technologies, they constitute our framework for secure identification of IoT devices as well as their owners. The steps leading a user, say, Alice, to get her smart thing an identity are as follows:

1. Alice logs into IdP service;
2. Selected Alice’s profile data (telephone number, email address, login name) are forwarded to IoT signing service;
3. IoT service generates a X.509v3 certificate for Alice’s watch and the corresponding TLSA entry, and sends them to Alice;
4. Alice adds TLSA record to DNS domain she manages;
5. Alice transfers the certificate to the device.

Here, we assume that the device communicates with outer world via TLS protocol, and has a public IP address. The latter assumption makes it possible to associate its name with IP address using DNS A record. The earlier one guarantees that the device will present its X.509 certificate (with IdP provided v3 extensions) to any connecting client. Note that like in the literature [3], the TLSA record stores an SHA256 certificate fingerprint rather than a whole certificate. This means that device owner data can be only be obtained by direct communication with that device, and are not stored elsewhere except IdP.

DNS DANE offers several modes of certificate matching. Following the literature [3], we switched off PKI validation and required users to calculate and match SHA256 for the whole certificate. Hence, the TLSA record “Usage”, “Selector”, and “Matching Type” field values of 3, 0, and 1, respectively.

A user, say, Bob, communicates with the device via TLS or Datagram TLS (DTLS) protocols and is presented the X.509 certificate with IdP v3 extensions. Then, he checks the certificate against the TLSA record with the use of any technology he trusts, for example, a set of existing programs (host, openssl). For convenience, we have bundled this functionality into a standalone program named IoT verifier. The verification process goes as follows:

- Bob enters the address of the device he wants to verify;
- IP address of the device gets resolved via DNSSec;
- A connection attempt is made to the device, which results in the device revealing its X.509v3 certificate;
- The certificate is checked against the TLSA record, and PKI trust chain for the certificate signature gets verified;
- Bob is presented IdP details about the device if verification is successful, or a verification error message.

The proposed framework does not pose any requirements about the strength of measures applied by an IdP to ensure identities of its users. Therefore, it can host variety of providers and technologies, ranging from highly secure, hardware-backed qualified digital signatures, to OpenID and authentication through social websites. Similarly, there can exist a number of IoT signing entities of all kinds: subsidiaries of IdPs, aggregators with agreements with many IdPs, subsidiaries of DNS registrars, and alike. This architecture can eventually host an ecosystem of IdPs and signing entities.

In our demo implementation, the official Polish identity provider service, named Trusted Profile (abbreviated PZ, for Profil Zaufany), was the IdP of our choice. PZ is the central user authentication service for the rest of the governmental administration services, enabling official document exchange with various state authorities by physical users as well as legal bodies. PZ user accounts are created on users’ requests; the users’ identities get confirmed by their online banking accounts, or by physical appearance at authorization points, like national post offices or city halls. This cost-free and convenient service has already won a substantial customer base of nearly 2 million registered users.

Moreover, PZ and the linked state services are able to interact with authorized third-party external systems. As a result, a number of dedicated services have appeared that accomplish specific processes, like university admissions. Our demo IoT signing service is one of them: it has been officially recognized as a trusted external system by the Center for Informatics Technology, the PZ operator, and has been granted permissions to use PZ as a Single Sign-On (SSO) service, as well as to retrieve basic user profile information.

**Figure 4:**
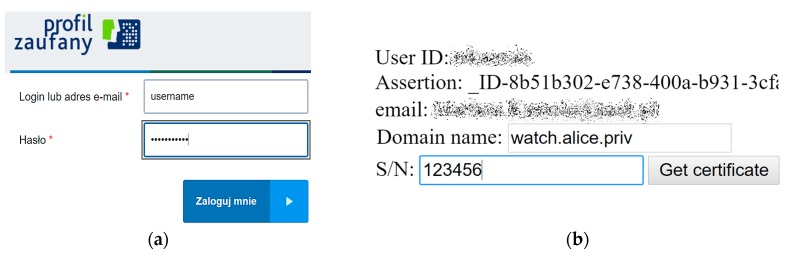
(**a**) PZ (Profil Zaufany) login form after redirection; (**b**) eGO-DANE certificate customization form.

Our demo IoT signing service is named eGO-DANE (eGO is the brand name for user identity provided by PZ). It runs with a public static IP address that is known to PZ service. A user fills simple HTML forms to interact with both services. On opening the eGO-DANE homepage, user browser is redirected to PZ login page (Step 1 in Figure 3). The login form is presented in Figure 4a. The redirection is made with eGO-DANE credentials, and after successful login, eGO-DANE is called back by PZ, and provided with user SAML assertion. This assertion is used to retrieve user profile data.

The user may choose which of his/her IdP data will be stored in the certificate. S/he can also provide some extra information to be stored, as shown in Figure 4b. Most of all, the requested domain name must be the one planned to be used to identify the device in the DNS zone maintained by the user. It will be stored in CN and subject alternative name URI fields, and checked while verifying the certificate. Other fields are considered auxiliary, for example, the serial number embedded in the certificate or any other data helping in physical identification of the device. The object identifier (OID) extension codes for X.509 certificate make it easy to expand the form with many more structured auxiliary data.

Once the form is complete, the user requests certificate generation, and is provided a certificate in PEM format, as well as the device private key and prepared TLSA record containing the certificate fingerprint. Now, the certificate and private key should be installed in the IoT device, and the TLSA record should be added to the proper DNS zone file.

The signing application has been developed in Java, and deployed to Apache Tomcat container running on a virtual Linux server (Debian 9, 1 virtual core, 4 GB RAM), provided by OVH SAS, Roubaix, France. DNS for test domains was also provided by OVH; it supports DNSSec, and zone management can be done either via web forms or by direct changes to zone file (apparently, it is impossible to manage DNS entries via some public programming interface). The project sources can be found in Supplementary Materials.

Developing the identity verification application has not placed such hard technology constraints, because it did not interact with any proprietary API. The application has been developed in Python using standard libraries for communication over TLS, DNS resolving, and certificate manipulation. The project sources can be found in Supplementary Materials.

Raspberry Pi Zero W by Raspberry Foundation, Cambridge, UK has been chosen as the intelligent device to be signed and checked in our scenario. At startup, it runs a simple Python HTTPS server—just enough to provide its certificate to any connecting client application. When Bob runs the identity verification program and enters the address of the Raspberry Pi device, he gets either a security identification failure message, or selected details of Alice taken from her PZ profile and stored in X.509 certificate. Eventually, Bob is able not only to verify the identity of Raspberry Pi, but also the identity of its owner—for example, by calling Alice on the phone number provided in the certificate.

Our choice to use Raspberry Pi in testbed was motivated by a rich suite of pre-installed applications that the device comes with. Script programming languages; decent processing power; built-in WiFi adapter, which can be set into access point mode—all that reduced risk of getting involved into hardware problems that could have been encountered in lower-grade appliances. With 512 MB RAM, Raspberry Pi Zero W definitely qualifies as Class 2, according to Internet Engineering Task Force (IETF) terminology [12], with native support for TLS and WiFi, which are the only compatibility criteria in our architecture.

Constrained devices are able to fulfill those compatibility criteria as well. It is reported [13] that Arduino, widely supported by IoT platforms, easily handles TLS with X.509 certificates. Examples of other compatible Class 2 devices are those listed by Amazon [14] and running FreeRTOS operating system, and some appliances running TinyOS on ARM, by Atmel [15]. With regards to Class 1 devices, there is evidence [16] of ARM appliance with 32 kB RAM and Contiki OS with X.509 support implemented. In the last case, it was found that transmitting a compressed certificate reduced the overall power consumption, and that the overhead for X.509 support was negligible.

## 3. Results

Performance evaluation results of the key components are presented in Table 1. In all cases, execution times for a sequence of calls to selected operations were recorded. The tests were run under good network conditions (at least 100 Mbps cable connection, or a WiFi connection in proximity of the device). The client and server machines were not busy with other user tasks.

Obtained latencies for all test cases are at acceptable levels for man-to-machine interaction. The total time for IdP query and making of X.509 certificate is well below 1 s. Response times from Raspberry Pi device vary widely, but still are below 1 s, too. DNS queries are fast and reliable because of caching. Applicability of the architecture to machine-to-machine scenarios would require more profound tests done in parallel, and depends heavily on the actual machine-to-machine case.

The architecture presented so far involves multiple entities in provision of a complete chain of authentication. Initially, Bob relies on DNSSec to be sure he is making a request on an authentic IP address of Alice’s watch. Security could be breached in this phase in the case where either an attacker had broken into Alice’s domain management system, or impersonated Alice while registering the domain. Those problems are commonly known and the proposed architecture is neutral to them.

Next, the device presents its X.509v3 certificate to Bob. Mechanisms of TLS and DNSSec + DANE make it virtually impossible for an attacker to forge the certificate, even if he had broken into Alice’s device. Finally, X.509v3 fields serve Bob for identification of the device owner. Data in those fields come from the publicly recognized identity provider, from the profile of the person that requested the certificate to be issued. Consequently, they correctly identify a person and, when joined by IoT signing service and DNS with IoT device data (domain name, key pair), they get stored inseparably in a certificate. As the IoT service is authenticated by PKI, there is no place where a security breach may occur, except from the hacking of either the IdP or the signing service.

We assume that in the above scenario, all parties are online, and the device has a public static IP address assigned. Such an assumption is unrealistic for simpler devices, like sensors or automation appliances. Even if they support IP communications, their addresses are local or dynamically assigned, or both. Our architecture can well support the case when the IoT device is offline, until it is capable of providing TLS or DTLS-based service, accessible locally. In such a situation, the client interested in identity verification cannot rely on DNS mapping between device domain name and IP address, because it is nonexistent. Yet, it can still use the rest of our architecture, provided s/he can assure identity of the device in some other safe way. We provide an option to store an extra serial number extension in the certificate. Its use depends on any naming agreement, for example, Vehicle Identification Number (VIN) number may be used to identify cars, as it can be found etched in spot places, and it is costly and risky to forge it well. For other devices, it can be engraved in similar way or, if possible, displayed on a device screen on user request. Note that such a visual identification procedure typically precludes fully automated IoT interaction, unless the device and client establish wired or any other assured channel for data exchange.

**Figure 5:**
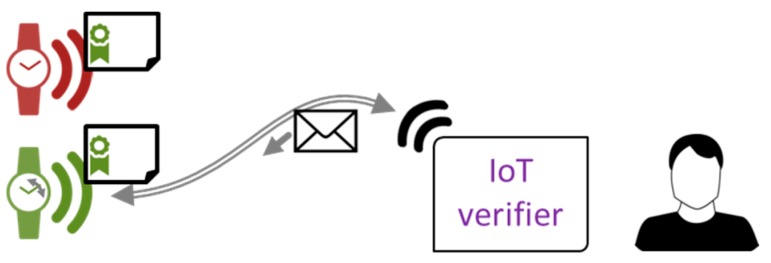
Physical identity verification for offline devices by user sending a nonce (shown as envelope). A malicious device (red) with stolen identity cannot know the number being sent.

If the device is offline and the user can connect only by wireless communication, which happens in most cases, we must provide another way to ensure he is interacting with the proper device, and not with its copycat containing a stolen key pair and certificate. We propose to enable the verifier program with an option to send a nonce (a number known only to the client) to the connected device, as shown in Figure 5. The device reports this number for example, by LED blinking the correct number of times. Such a method was implemented in our demonstration software; in the case of Raspberry Pi Zero, power diode was used (the only factory-built one in this model). Other devices may use other nonce display methods; consider Alice’s watch wagging its second hand the appropriate number of times, for example. If the user gets connected to the malicious device, the original one does not react to the nonce being sent. On the opposite hand, if the malicious device wants to simulate the original one, the chances of guessing the nonce and displaying it in proper timing are low and can be reduced further by replaying this scenario.

The scenario described until now in this chapter is a base one, covering both device and device owner identity authentication. Its variation for offline devices has also been considered. The proposed architecture relies as much as possible on existing technologies (DNSSec, PKI, TLS, phone calls, text messages, and visual identification), which makes it capable to be applied in various scenarios. Below, we provide a few of them.

### 3.1. Change of Ownership

This is the base scenario continued: once Bob has made sure of the identities of watch and of Alice, and that Alice is the watch owner, he may want to buy that smart device. In order to comply with our architecture, he must have an account with any of IdPs supported by any IoT signer service (we assume that many entities of both kinds operate on the market). He also needs to administer any domain with DNSSec enabled, in order to create resource records for the device being bought. He is able, as any other person, to use the IoT signer to obtain a certificate with his credentials, and to prepare DNS TLSA and A records. All those preparations are, however, void until Alice hands over administrative privileges for the watch to Bob, allowing for final transfer of his certificate into the device.

The procedure of watch trade has been completed but due to DNS caching, it may take some time before Bob appears to everyone as the new owner. This lag can be shortened by reduction of TTL (time-to-live) parameters for the DNS records or, and probably better, by Bob following certification procedure some time in advance, if possible.

### 3.2. Lease

This case covers many scenarios where the device is handed away temporarily, for example, in car rental, home appliances repair, sports equipment rental and service, circulation of containers in supply chain, pet medical treatment, and the like. Here, the device owner authorizes a tenant to execute partial control of various scope, however, the device certificate does not change. It is DNS that is used as the only storage of the lease contract details. Such an approach was successfully applied by other architectures: sender policy framework, DNS-service discovery, some email antivirus scanners, and so on. They mostly used TXT resource record; similarly, we propose that Canonical Name (CNAME) record will be used by the borrower and TXT record will be used by the lender.

We postulate that the borrower creates a DNS name for the device being taken under his/her care. The borrower creates a CNAME record for that name, which redirects requests to the real owner DNS records. This is understood as a unilateral declaration “I tend the device CNAME record points to”. Reciprocal confirmation of such a statement must be expressed by the lender in his/her TXT record. TXT record type specification was created to store any extra domain related information in human readable form and, although the *key* = *value* description convention is a de facto standard, there is no officially recognized key dictionary that would suit the lender to describe the contract details. Consequently, in our architecture, Alice, who now lends her watch to Bob, would prepare a TXT record as follows: 443._tcp.watch IN TLSA TXT “leased-under-name = watch.bob.priv” or more TXT records with contract details, like territorial or subleasing constraints, according to some agreed metadata scheme.

Note that because of DNS limitations for CNAME usage, only the device owner is eligible to provide TXT record with contract details.

### 3.3. Related IoT Devices

**Figure 6:**
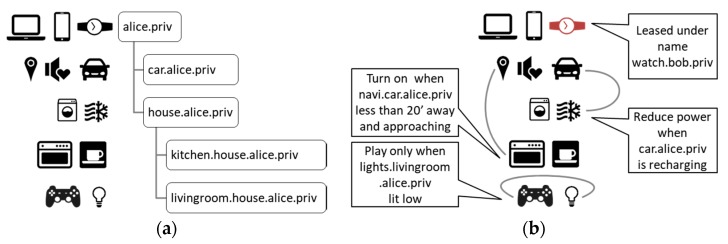
(**a**) Hierarchical device organization by DNS naming; (**b**) Introducing custom relationships by annotations in DNS TXT resource records.

DNS records may serve as well to define relationships between different IoT devices that have representation in their domain names. Hierarchical relationships within a single domain are interpreted naturally using DNS naming hierarchy itself. Alice may establish a naming scheme for her stuff, for example, by its location as shown in Figure 6a, where devices are given names within domains on the same line. Such implementation may raise doubts about Alice’s privacy; although DNS does not reveal domain names structure, replacing self-explanatory names with random ones (e.g., Universally Unique Identifiers, UUIDs) could be a good precaution.

Independent of those natural hierarchical naming capabilities, DNS TXT resource records can serve for defining any other custom relationships between devices, as shown in Figure 6b. In our example, they take the form of asymmetric rules for device activities. Alice may introduce them for the sake of environment care (car vs. HVAC operation), her health (gaming/lighting), her comfort (car location/food heating), and many others. Once the rules are stored in machine readable format, they can influence Alice’s lifestyle and determine household operations. The same approach can be applied in industry, and thanks to DNS distributed architecture, it would not adversely affect its efficiency and reliability.

## 4. Discussion

The given proposition of architecture tries to make the best use of the existing DNS system as a repository of smart device identities and relationships, by introduction of as few additional new technologies as possible. In this regard, it is similar to ideas already described [3,4,5,7,8,9,10,11]. Our contribution is to involve the existing range of identity providers in the process of X.509 certification. Eventually, it will lead to an ecosystem of IdP proxies providing domain owners a convenient way to generate and store TLSA records. Such functionality can be also provided directly by IdPs or by domain registrars, as an additional service. The main goal is to deliver an attractive and open alternative for today’s proprietary IoT management platforms. The architecture imposes modest technical requirements on the devices, which basically amount to the support of TLS with X.509 certificate. This is much in line with what current IoT management platforms want [13]. For example, the Azure IoT platform maintains a list of over 1000 certified compatible devices—all of them are Class 2, cf. for example, [17]. However, it has been proved [16] that Class 1 constrained devices, present in Wireless Sensor Networks (WSN), can also support X.509 certificates.

There is no reason to expect scalability of such a system to be inferior to scalability of the DNS system on which it is based—and the latter one is known to operate very efficiently. The delay of DNS record updates visibility can in turn be controlled to some extent by TTL and caching policies adjusting.

**Figure 7:**
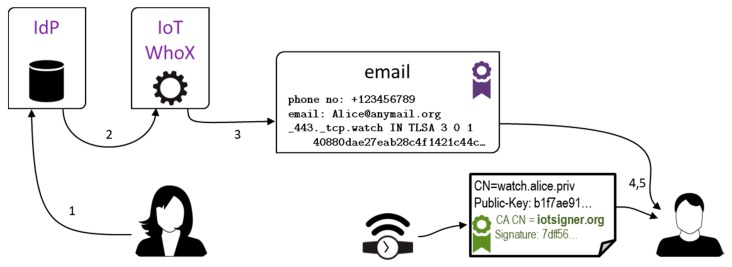
Selective communication of personal information.

The ultimate concern that can arise about this technology is privacy. Like for WHOIS data, exposing extra information publicly is generally not an issue for institutional device owners, but it effectively scares away individuals. Device owners must be provided means to reveal their sensitive data only to selected parties. We consider giving devices names that are hard to guess not an option if the owner really cares for its privacy. Instead, we propose to implement an additional service, here named WhoX, which runs much the same way as the IoT signer, see Figure 7 and compare with Figure 3. Alice authenticates herself with the IdP, and provides the email address of Bob, to whom she wants her email and phone number to be sent. WhoX composes a signed message to Bob, carrying Alice’s data retrieved from IdP. Bob is provided with Alice’s extra contact data. Note that in this case, Alice’s watch X.509v3 certificate does not have to contain her contact data directly.

The last issue to address is how to accomplish storage of ownership information for things incapable of emitting X.509v3 certificate, but already given their unique and persistent IDs in some other way. Beacons, computer hardware components, Class 0 sensors, or any non-electronic devices with their serial numbers securely attached are examples of such appliances. Their association with owners can also be accomplished in DNS records. Unlike previously, all devices with the same ID type must be given names equal to their ID values, and placed in the same globally known domain. Thus, all IBAN (international bank account number) identifiers whose owners want to advertise via DNS could be either in domain iban.org, or in national domains like iban.org.de, iban.org.pl, and so on. To prevent identity theft, those domains would have to be managed by appropriate authorities, and coupled with branch-related registers. Owner authentication procedure would, in general, follow that in Figure 3, but with many branch IdPs involved.

In summary, we have shown that the idea of using DNS to store smart device owners’ identities is desirable and technically viable. Increasing trust between smart devices and human users by providing device owner identity will stimulate creation of new interaction scenarios, for example:

- a buyer or leaser can check authenticity or ownership of an item;
- some device may provide its services only to clients whose identities are confirmed by IdP;
- devices may team up and exchange sensitive data only when their owners’ identities are confirmed.

Operation of such architecture in a testbed using real DNS and IdP systems has been proven.

Furthermore, if DNS TXT records get employed to store relationships, additional scenarios are possible:

- when a device is temporary leased or serviced, parties can declare this fact mutually;
- multiple complex rules of interaction between devices can be written to a number of TXT records.

Possibilities of machine interpretation of TXT records data depend only on existence of some common taxonomy of terms used. Although the specification recommends the *key* = *value* format, any other one can be used, e.g., Resource Description Framework (RDF), like proposed in the literature [18].

## Figures and Tables

**Table 1:** Real execution times for selected operations, measured by the calling code [msec]. IdP—identity provider.

Operation	Minimum	Maximum	Mean	Standard Deviation	No. of Samples
Get user info from IdP	243	384	258	15	100
Generate X.509 certificate with user data	64	470	127	98	25
Get X.509 certificate from Raspberry Pi	29	586	38	55	100
Query DNS for TLSA record ^1^	7.7	26	11	3	100

^1^ Using default DNS resolver in Windows 10 Professional.

## Supplementary Materials

The source code for IoT signer and verifier programs is available online at http://www.mdpi.com/1424-8220/18/8/2517/s1.
