# Supplementary material for: Internet of Things with Lightweight Identities Implemented Using DNS DANE—Architecture Proposal
Source: Sensors (Basel). 2018 Aug 1;18(8):2517. doi: 10.3390/s18082517 (PMC6111735; doi:10.3390/s18082517)
Supplement: Supplementary file 1 [file sensors-18-02517-s001.zip › figures/4.pdf]

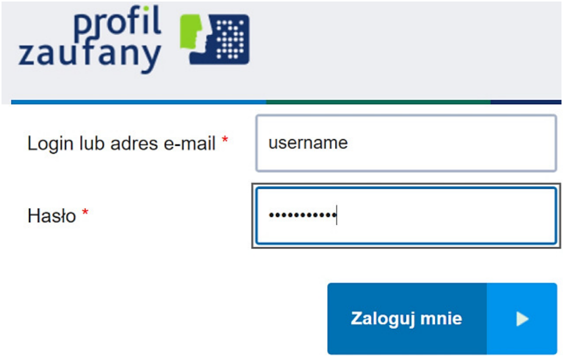
profil zaufany
Login lub adres e-mail *
username
Hasło *
Zaloguj mnie

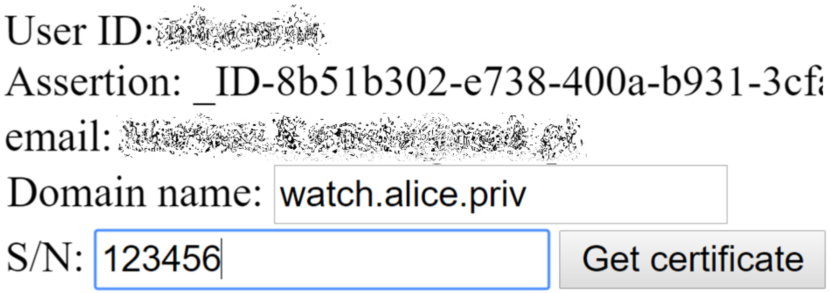
User ID:
Assertion: _ID-8b51b302-e738-400a-b931-3cf
email:
Domain name:
watch.alice.priv
S/N:
123456
Get certificate
