# Supplementary figures and images for: Internet of Things with Lightweight Identities Implemented Using DNS DANE—Architecture Proposal
Source: Sensors (Basel). 2018 Aug 1;18(8):2517. doi: 10.3390/s18082517 (PMC6111735; doi:10.3390/s18082517)

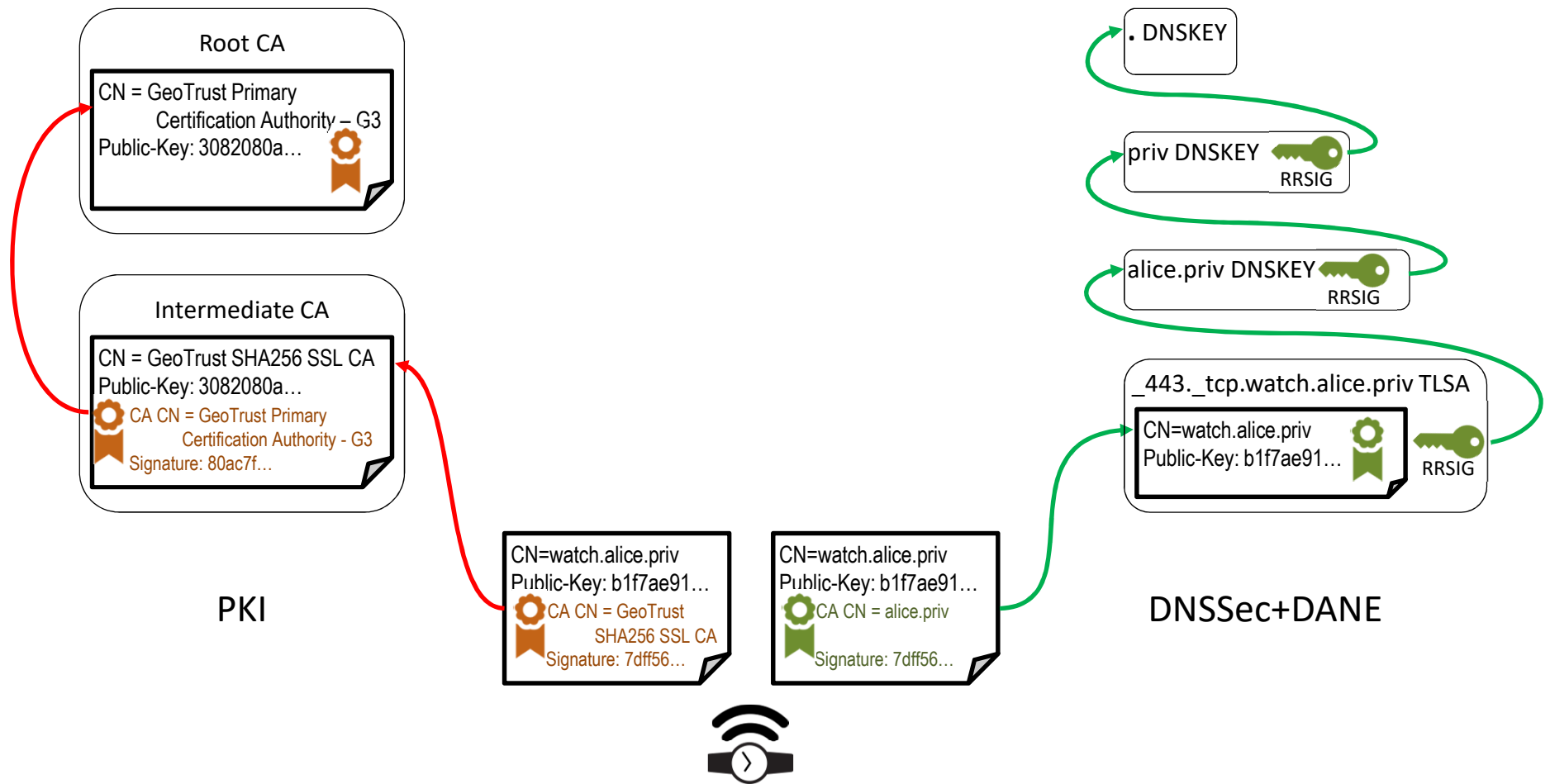

Supplement: Supplementary file 1 [file sensors-18-02517-s001.zip › figures/1.pdf]

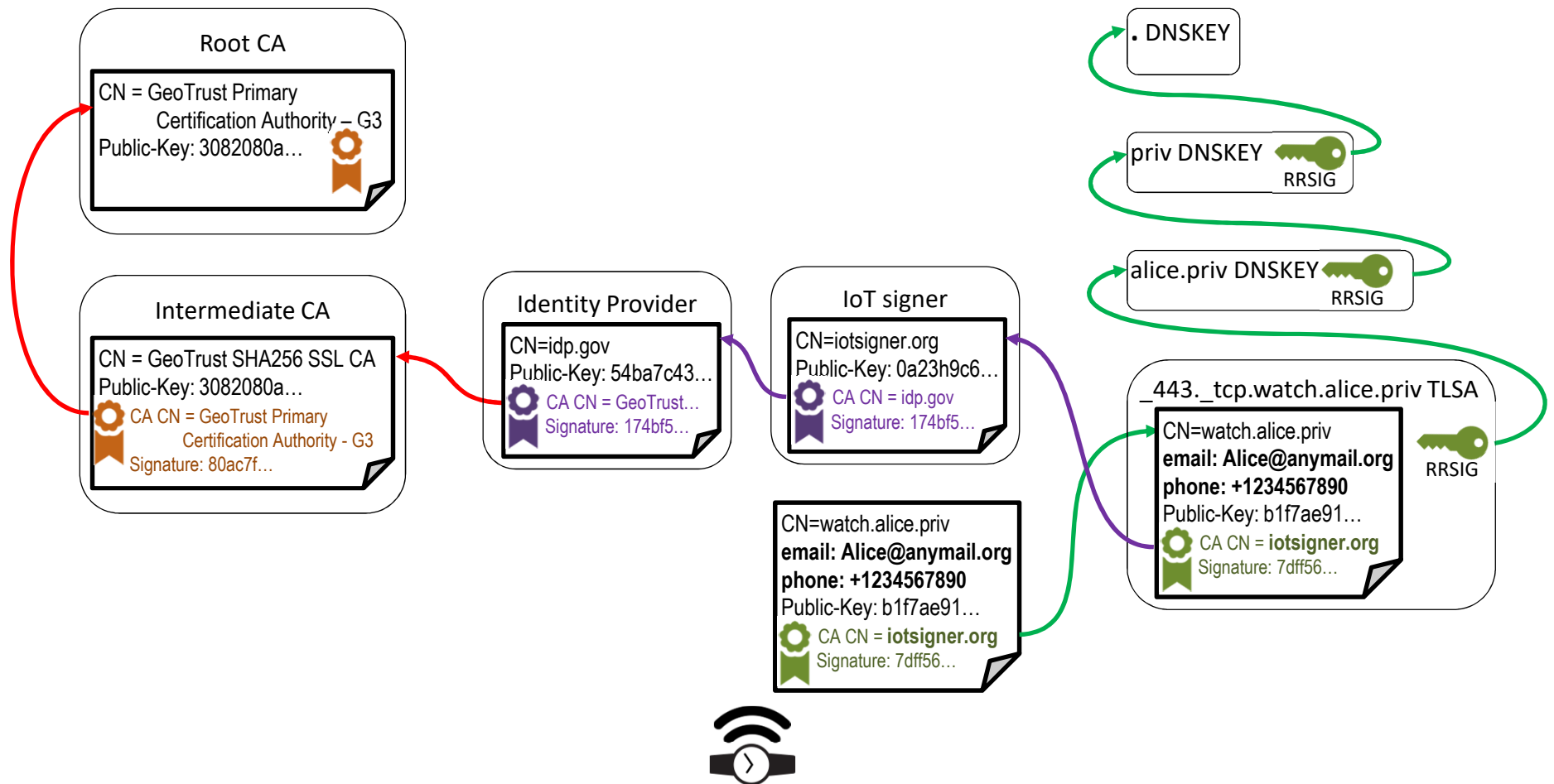

Supplement: Supplementary file 1 [file sensors-18-02517-s001.zip › figures/2.pdf]

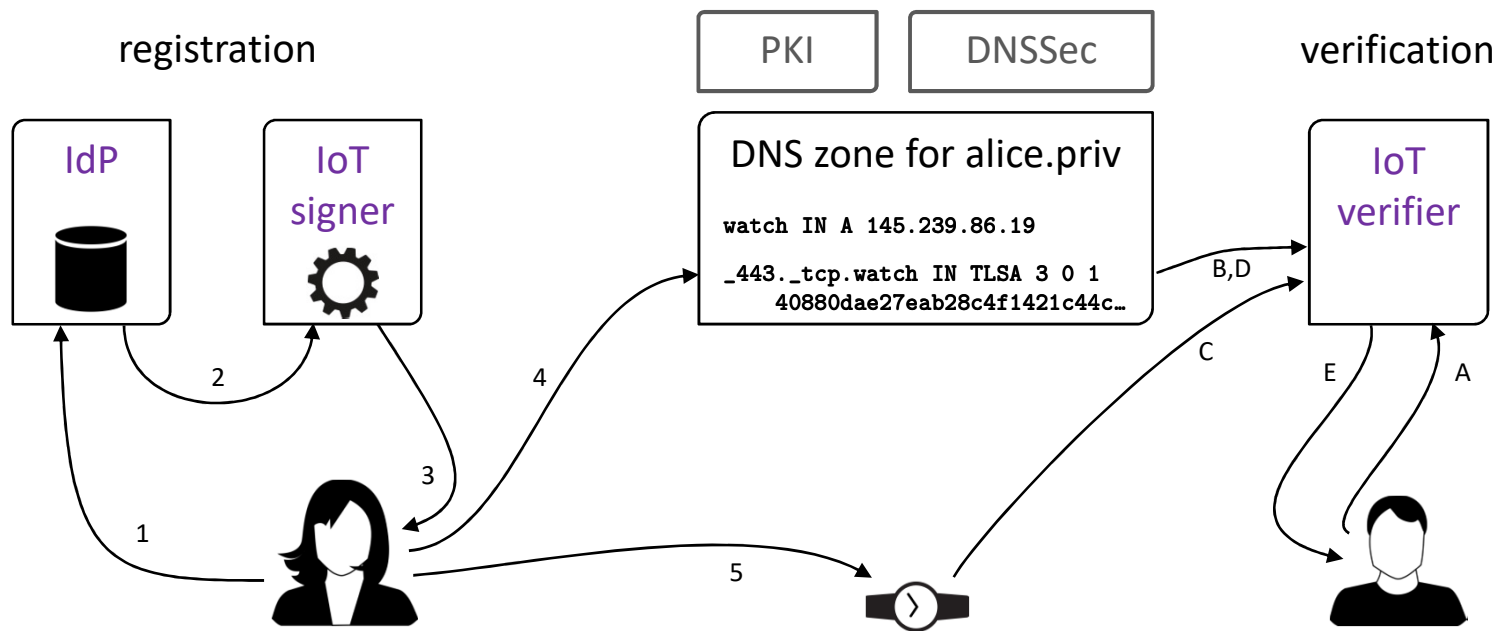

Supplement: Supplementary file 1 [file sensors-18-02517-s001.zip › figures/3.pdf]

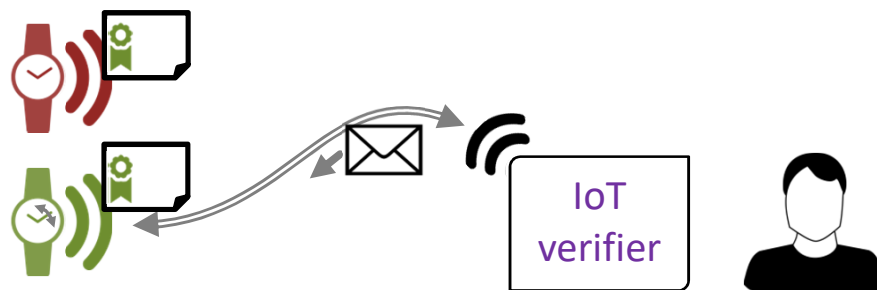

Supplement: Supplementary file 1 [file sensors-18-02517-s001.zip › figures/5.pdf]

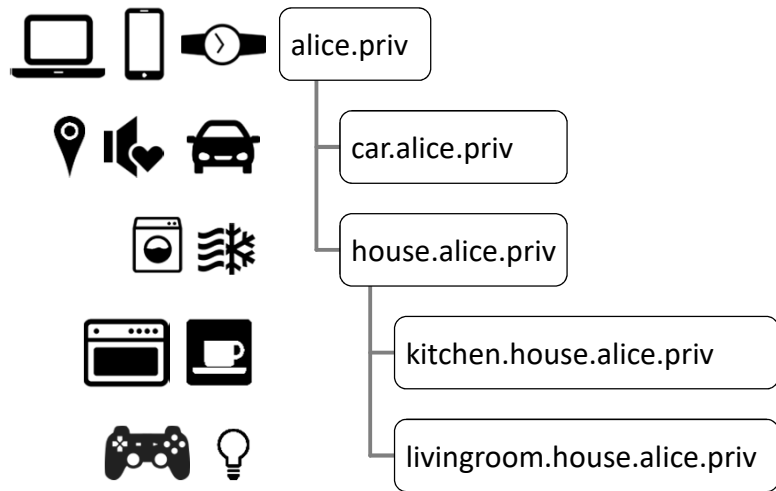

Supplement: Supplementary file 1 [file sensors-18-02517-s001.zip › figures/6a.pdf]

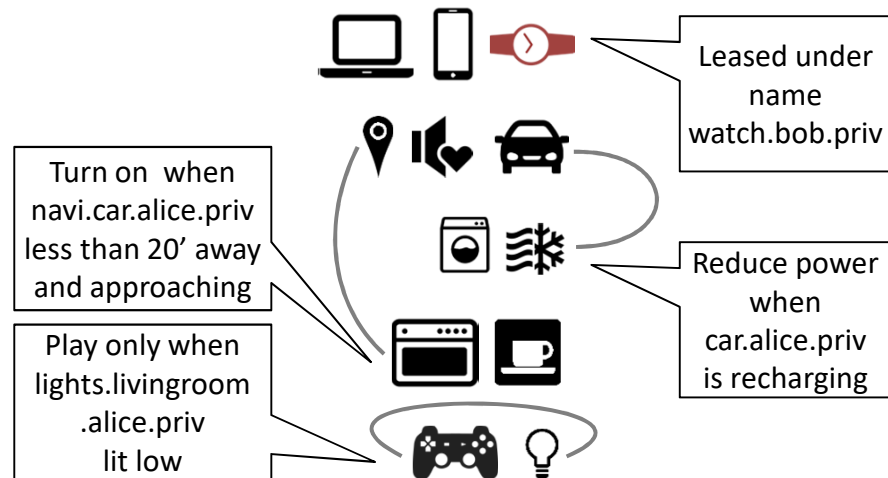

Supplement: Supplementary file 1 [file sensors-18-02517-s001.zip › figures/6b.pdf]

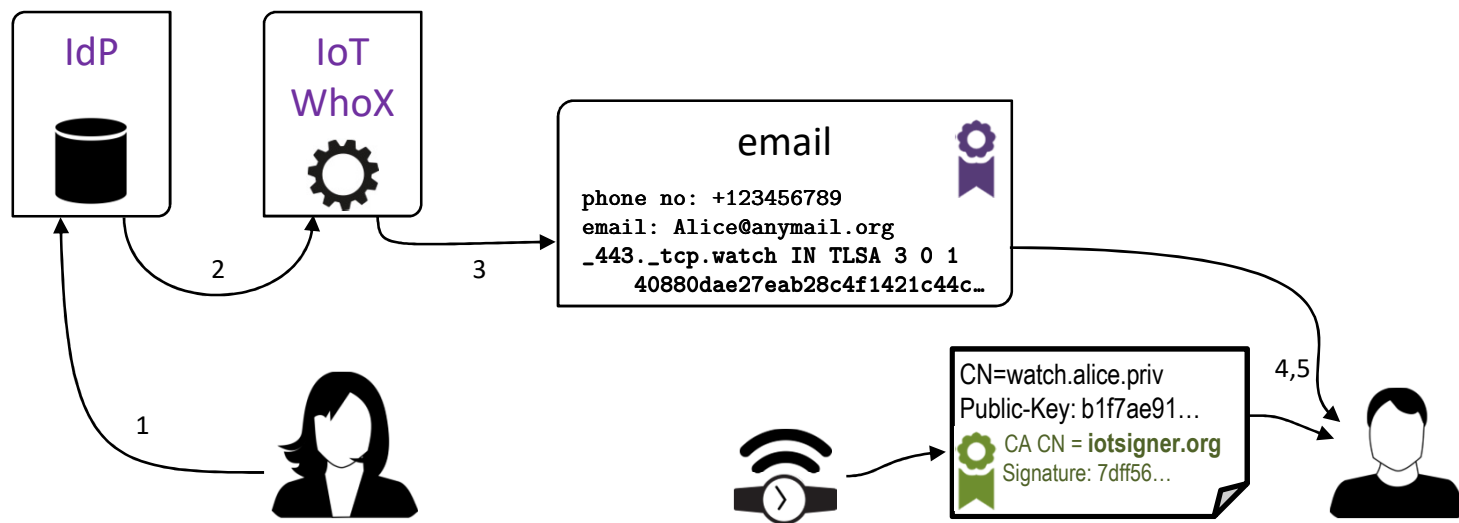

Supplement: Supplementary file 1 [file sensors-18-02517-s001.zip › figures/7.pdf]
